# Aldehyde Dehydrogenase 2 (*ALDH2*) Genotype Affects Rectal Cancer Susceptibility Due to Alcohol Consumption

**DOI:** 10.2188/jea.12.70

**Published:** 2007-11-30

**Authors:** Keitaro Matsuo, Nobuyuki Hamajima, Takashi Hirai, Tomoyuki Kato, Kouichi Koike, Manami Inoue, Toshiro Takezaki, Kazuo Tajima

**Affiliations:** 1 Division of Epidemiology and Prevention, Aichi Cancer Center Research Institute.; 2 Department of Gastroenterological Surgery, Aichi Cancer Center Hospital.; 3 Department of Clinical Laboratory, Aichi Cancer Center Hospital, 1-1 Kanokoden, Chikusa-ku, Nagoya 464-8681.; 4 Department of Epidemiology, Nagoya University Graduate School of Medicine, 65 Tsurumia-cho, Nagoya 466-8550.

**Keywords:** colorectal cancer, genetic predisposition of disease, alcohol, *ALDH2* polymorphism

## Abstract

Background: Epidemiologic studies have shown the association between alcohol consumption and colorectal cancer, especially for rectal cancer. The alcohol related enzyme encoding gene *ALDH2* has polymorphism Glu487Lys, and 487Lys allele is closely linked with phenotypic loss of enzyme activity. Materials and Methods: A hospital-based case-control study was conducted with 72 colon and 70 rectal cancer cases and 241 non-cancer controls to evaluate the alcohol consumption and *ALDH2* Glu487Lys polymorphism. The logistic regression model was applied to estimate the odds ratios (ORs). Result: The crude ORs for *Glu/Lys* and *Lys/Lys* genotype relative to *Glu/Glu* for colon and rectal cancer were not statistically significant. However, with the rectal cancer analysis, the ORs for high alcohol consumption were greater with *487Glu/Lys* genotype compared with *Glu/Glu*, albeit not. Conclusions: These observations suggested rectal cancer risk might be influenced by *ALDH2* gene polymorphism. The prevention effect by alcohol reduction might differ by *ALDH2* genotype.

## INTRODUCTION

Since the first pioneering study in England and Wales in 1957^1^^)^, many epidemiological investigations have been conducted to determine whether an association exists between alcohol and risk of colorectal cancer^2^^,^^3^^)^. These studies suggest that alcohol consumption increases the risk, and a positive association has been more consistently observed for cancer of the rectum^3^^)^.

Although several mechanisms can be hypothesized concerning alcohol effects, mucosal stimulation due to the metabolite, acetaldehyde, is regarded as of particular importance. Acetaldehyde bind with macromolecules and proteins thus forming acetaldehyde adducts^4^^,^^5^^)^ which can interfere with DNA repair activity^6^^)^ and can act as neoantigens with a subsequent immune response^7^^)^. In the rat experiments, acetaldehyde causes hyperproliferation of rectal mucosal tissue^8^^,^^9^^)^. A similar mechanism is also proposed for colonic mucosa, but the experimental evidence for this is lacking. Only one study showed the hyperregeneration of rectal mucosa in alcoholics, which indicating high exposure to alcohol and its metabolite may induce regeneration leading to carcinogenesis^10^^)^. Although it needs further experimental studies, epidemiological evidence is consistent with the effect of acetaldehyde on rectal mucosa observed in rats.

Aldehyde dehydrogenase-2 (ALDH2) is a tetrameric enzyme which generates acetic acid from acetaldehyde and whose activity correlates with the in vivo concentrations of this derivative of alcohol^11^^)^. A polymorphism of *ALDH2* (Glu487Lys) is prevalent in Asians^12^^)^, and the *487Lys* allele is closely linked with phenotypic loss of enzyme activity. This polymorphism is located in a small three-stranded β-sheet domain that acts as an interface for tetramer formation and substitution with Lys at codon 487 affects the status of the ALDH2 enzyme^13^^)^. Several of studies showed the correlation between phenotype such as drinking behavior *I* alcohol related symptoms and three genotypes by this polymorphism. The activity decreases in the order, *Glu/Glu*, *Glu/Lys*, and *Lys/Lys*^14^^,^^15^^)^. Thus, there is a distinct possibility that the *ALDH2* polymorphism could impact on the risk of colorectal cancer. Two studies in Japan have already examined this question, and both pointed to risk elevation in subjects having the ‘weak allele’^16^^,^^17^^)^. Nevertheless, the gene-environment interaction between alcohol consumption and the *ALDH2* genotype has not hitherto been detailed, to our knowledge. We therefore conducted a hospital based case-control study at Aichi Cancer Center (ACC).

## METHODS

### Study population and sample collection.

The case-control study was conducted as a part of series in a major project on genetic polymorphisms and cancer risk with patients at ACC^18^^)^. Cases were recruited who were confirmed histologically to have colon (n=72) or rectal (n=70) cancer, excluding those with a history of other types of malignancies. Controls (n=241) were outpatients without any history of cancer who visited ACC during March to December 1999 for gastroscopy^19^^)^; including 97 (40.2% out of 241) participants stated to be under medication for 107 diseases (not confirmed by their medical records); 23 with gastric/duodenal ulcer, another 23 for so-called gastritis, 16 with hypertension, 8 for pain including arthritis and lumbago, 7 with diabetes mellitus, 7 with hyperlipidemia, and other 23 miscellaneous diseases. All cases and controls were Japanese. The subjects who provided written informed consent for participation in this study were asked to complete a self-administered questionnaire and to provide blood from a peripheral vein. This study was approved by the Ethical Committee of ACC (Approval No. 12-23 and 12-27).

### Environmental factors

Alcohol drinking was divided into three categories based on mainly drinking quantity; low (less than once a week), moderate (once a week or more frequently with less than 50mL of ethanol) or high (once a week or more frequently with 50mL of ethanol or more). Smokers were also divided into three categories (never, former, and current). We defined former smokers as those who had quit smoking more than 2 years before disease onset or the questionnaire study. For dietary factors, we asked frequency for four food items (whole meat, fish, raw vegetable, and tofu), three beverages (Japanese tea, black tea, and coffee), and preference for salt. We asked the cases to provide information about their life-style before the onset of disease, and the controls at the study enrollment.

### Genotype analyses of the ALDH2

DNA of each subject was extracted from the buffy coat fraction with a QIAamp DNA Blood Mini Kit (Qiagen Inc., Valencia, CA). *ALDH2* 1543 G to A (accession no. NM_000690, Glu487Lys) was genotyped using PCR-CTPP (polymerase chain reaction with confronting two-pair primers) method developed in our laboratory^20^^)^, as previously described^21^^)^.

### Statistical analysis

Accordance with the Hardy-Weinberg equilibrium (HWE), which indicates an absence of discrepancies between genotype and allele frequencies, was checked for controls with a χ^2^ test. Categorical variables were also tested in the same way. All odds ratios (ORs) and 95% confidence intervals (CIs) were estimated by an unconditional logistic regression model for each site of cancer and their combination. Age was adjusted as a continuous variable. All of the statistical analyses in this study were performed using STATA version 7.0 statistical software (STATA Corporation Inc., College Station, TX). Adjustment for multiple comparison was not performed, because the analyses were conducted in an exploratory context^22^^)^, which requires a careful interpretation of any p-values.

## RESULTS

A total of 142 cases and 241 controls were recruited. Their characteristics are shown in Table 1. Cases comprise 72 colon cancer and 70 rectal cancer patients. The age distribution was slightly older among colon cancer patients. The percentage of current male smokers was slightly higher in those with rectal cancer, but the distribution of smoking status was not statistically significant by χ^2^ test. High alcohol consumers were predominant in male rectal cancer cases with statistical significance (χ^2^ = 41.1, p <0.001).

**Table 1.** Background characteristics of cases and controls by sex.

	Males						Females					
	
	Controls		Cases				Controls		Cases			
			
			Colon		Rectum				Colon		Rectum	
	N=118		N=42		N=41		N=123		N=30		N=29	
Number (%) of												
Age in years												
<40	2	(1.7)	3	(7.1)	0	(0)	0	(0)	2	(6.7)	2	(6.9)
40-49	21	(17.8)	7	(16.7)	6	(14.6)	23	(18.7)	2	(6.7)	5	(17.2)
50-59	34	(28.8)	12	(28.6)	16	(39.0)	56	(45.5)	12	(40.0)	9	(31.0)
60-69	61	(51.7)	12	(28.6)	15	(36.6)	44	(35.8)	8	(26.7)	10	(34.5)
>70	0	(0)	8	(19.1)	4	(9.8)	0	(0)	6	(20.0)	3	(10.3)
Years from diagnosis												
≤ 3 year	-		26	(61.9)	30	(73.2)	-		21	(70.0)	24	(82.8)
> 3 year	-		16	(38.1)	11	(26.8)			9	(30.0)	5	(17.2)
Smoking status												
Never	34	(28.8)	14	(33.3)	4	(9.8)	106	(86.2)	27	(90.0)	26	(89.7)
Former	38	(32.2)	18	(42.9)	18	(43.9)	5	(4.1)	1	(3.3)	0	(0.0)
Current	46	(39.0)	10	(23.8)	19	(46.3)	12	(9.8)	2	(6.7)	3	(10.3)
Drinking status												
Low (< 1 time /week)	35	(29.7)	9	(21.4)	6	(14.6)	101	(82.1)	26	(86.7)	23	(79.3)
Moderate*^1^	50	(42.3)	17	(40.5)	12	(29.3)	17	(13.8)	3	(10.0)	5	(17.2)
1-4 times /week	22		8		3		12		1		3	
≥ 5 times /week	28		9		9		5		2		2	
High*^1^	33	(28.0)	16	(38.1)	23	(56.1)	4	(3.3)	1	(3.3)	1	(3.5)
1-4 times /week	8		3		1		3		0		1	
≥ 5 times /week	25		13		22		1		1		0	
Unknown	0	(0)	0	(0)	0	(0)	1	(0.8)	0	(0)	0	(0)

*^1^ ‘Moderate’ indicates once per week or more frequently with less than 50 mL of ethanol and ‘High’ indicates once per week or more frequently with more than 50 mL.

The frequency of *ALDH2 Glu/Glu*, *Glu/Lys* and *Lys/Lys* genotypes were 52.3%, 39.8% and 7.9%, respectively for controls in total. The χ^2^ test for Hardy-Weinberg equilibrium with the polymorphism was not statistically significant (χ^2^ =0.014, p=0.905), indicating that no selective mechanisms for a specific genotype existed among the controls. When subjects were divided by sex, genotype distributions for total subjects or each site were not statistically different between males and females. The tests for HWE were not statistically significant for each sex, either (Table 2). The genotype frequencies for cases in total were 66.7%, 30.5%, and 2.8% for colon cancers, and 47.1%, 45.7%, and 5.7% for rectal cancers, the distribution among each site not differing significantly from that of controls. The ORs for *ALDH2 Glu/Lys* and *Lys/Lys* genotypes were not statistically significant. Point estimates for *ALDH2* ORs showed a tendency for risk reduction with the *487Lys* allele for colon (crude ORs compared with *Glu/Glu: Glu/Lys* 0.48 and *Lys/Lys* 0.23 for males; *Glu/Lys* 0.83 and *Lys/Lys* 0.36 for females), but not for rectal cancer (crude OR: *Glu/Lys* 1.07 and *Lys/Lys* 0.66 for males; *Glu/Lys* 1.71 and *Lys/Lys* 1.11 for females).

**Table 2.** Number of Cases and Controls, Odds Ratios (OR) and 95% CIs, for *ALDH2* polymorphisms.

Genotype	Males						Female					
	
	Controls	Cases	Model*^2^ 1		Model 2		Controls	Cases	Model 1		Model 2	
							
			OR	95%CI	OR	95%CI			OR	95%>CI	OR	95%CI
All cases	N=118	N=83					N=123	N=59				
Glu/Glu	65(55.1%)	53(63.9%)	1.00	Reference	1.00	Reference	61(49.6%)	28(47.5%)	1.00	Reference	1.00	Reference
Glu/Lys	44(37.3%)	26(31.3%)	0.72	0.40-1.33	0.70	0.38-1.30	52(42.3%)	28(47.5%)	1.17	0.62-2.23	1.11	0.58-2.14
Lys/Lys	9(7.6%)	3(3.6%)	0.41	0.11-1.59	0.38	0.10-1.51	10(8.1%)	3(5.1%)	0.65	0.17-2.56	0.63	0.16-2.48
UK*^1^	0(0.0%)	1(1.2%)	-				0(0.0%)	0(0.0%)				
Colon cancer	N=118	N=42					N=123	N=30				
Glu/Glu	65(55.1%)	31(73.8%)	1.00	Reference	1.00	Reference	61(49.6%)	17(56.7%)	1.00	Reference	1.00	Reference
Glu/Lys	44(37.3%)	10(23.8%)	0.48	0.21-1.07	0.47	0.21-1.06	52(42.3%)	12(40.0%)	0.83	0.36-1.89	0.78	0.33-1.83
Lys/Lys	9(7.6%)	1(2.4%)	0.23	0.03-1.92	0.22	0.03-1.85	10(8.1%)	1(3.3%)	0.36	0.04-3.00	0.38	0.05-3.30
Rectal cancer	N=118	N=41					N=123	N=29				
Glu/Glu	65(55.1%)	22(53.7%)	1.00	Reference	1.00	Reference	61(49.6%)	11(37.9%)	1.00	Reference	1.00	Reference
Glu/Lys	44(37.3%)	16(39.0%)	1.07	0.51-2.27	1.11	0.51-2.38	52(42.3%)	16(55.2%)	1.71	0.73-4.00	1.61	0.26-4.31
Lys/Lys	9(7.6%)	2(4.9%)	0.66	0.13-2.27	0.65	0.12-3.35	10(8.1%)	2(6.9%)	1.11	0.21-5.77	1.03	0.20-5.39
UK	0(0.0%)	1(2.4%)	-				0(0.0%)	0(0.0%)				

*^1^ UK indicates *ALDH2* genotype unknown because DNA was not amplified by PCR. This subject was excluded from OR analyses.

*^2^ Model 1: crude OR. Model 2: adjusted for age and smoking status.

Alcohol consumption was not adjusted because of strong confounding with *ALDH2* genotype.

Table 3 shows the ORs for the alcohol consumption according to *ALDH2* genotype. Among individuals with *ALDH2 Glu/Glu* genotype, high alcohol consumption was linked with a high OR for rectal cancer (OR 3.41, 95% CI 0.92-12.6). The OR for high alcohol consumption was also higher than unity for colon cancer, but to a lesser extent. Similarly, a significantly high OR was observed among rectal cancer with *Glu/Lys* genotype (OR 8.07, 1.88-34.7), whereas no significant OR for colon cancer. Although ORs lower than unity were obtained for the moderate or high alcohol consumption among colon cancer cases, the numbers were limited in these groups compared with rectal cancer. Focusing on *ALDH2 Lys/Lys* type, there were no high alcohol consumption subjects. Although the OR for moderate alcohol consumption was extremely high for rectal cancers with *Lys/Lys* genotype, the number of cases in this group was limited. When focusing on males, similar trends were observed that the OR for high alcohol consumption among rectal cancer. The OR for males highly consuming alcohol with *Glu/Glu* genotype was 4.00 (0.45-35.5), whereas that with those with *Glu/Lys* genotype was 13.2 (2.24-78.0). On the other hand, analyses for females showed unstable estimation that might be due to small number of alcohol consumers among females.

**Table 3.** Adjusted*^1^ ORs and 95% CIs for alcohol drinking according to *ALDH2* genotype.

Alcohol consumption	Controls	All cases			Colon cancer			Rectal cancer		
		
			OR	95%CI		OR	95%CI		OR	95%CI
Total	N=240*^2^	N=141*^3^			N=72			N=69*^3^		
*ALDH2* 487 *Glu/Glu*										
	N=126	N=81			N=48			N=33		
Low	54(42.9%)	25(30.9%)	1.00	Reference	17(35.4%)	1.00	Reference	8(24.2%)	1.00	Reference
Moderate	40(31.7%)	24(29.6%)	1.15	0.51-2.63	15(31.3%)	0.94	0.35-2.47	9(27.3%)	1.53	0.46-5.13
High	32(25.4%)	32(39.5%)	1.92	0.77-4.80	16(33.3%)	1.21	0.41-3.52	16(48.5%)	3.41	0.92-12.6
		P for trend:	0.138			0.693			0.050	
*ALDH2 487 Glu/Lys*										
	N=95	N=54			N=22			N=32		
Low	64(67.4%)	35(64.8%)	1.00	Reference	17(77.3%)	1.00	Reference	18(56.3%)	1.00	Reference
Moderate	26(27.4%)	10(18.5%)	0.76	0.30-1.98	4(18.2%)	0.53	0.14-1.97	6(18.8%)	1.08	0.33-3.54
High	5(5.3%)	9(16.7%)	3.59	0.99-13.0	1(4.6%)	0.69	0.07-6.89	8(25.0%)	8.07	1.88-34.7
		P for trend:	0.155			0.436			0.013	
*ALDH2* 487 *Lys/Lys*										
	N=19	N=6			N=2			N=4		
Low	18(94.8%)	4(66.7%)	1.00	Reference	1(50.0%)	1.00	Reference	3(75.0%)	1.00	Reference
Moderate	1(5.2%)	2(33.3%)	24.5	0.77-786.7	1(50.0%)	18.2*^4^	0.58-569.7	1(25.0%)	7.44	0.22-256.1
High	0(0.0%)	0(0.0%)	NE*^3^		0(0.0%)	NE*^5^		0(0.0%)	NE*^5^	
		P for trend:	0.070			NE*^4^			0.744	

Male	N=118	N=82*^3^			N=42			N=40*^3^		
*ALDH2* 487 *Glu/Glu*										
	N=65	N=53			N=31			N=22		
Low	8(12.3%)	5(9.4%)	1.00	Reference	4(12.9%)	1.00	Reference	1 (4.5%)	1.00	Reference
Moderate	28(43.1%)	18(34.0%)	1.04	0.29-3.70	12(38.7%)	0.88	0.22-3.49	6(27.3%)	1.68	0.18-16.2
High	29(44.6%)	30(56.6%)	1.69	0.49-5.84	15(48.4%)	1.07	0.27-4.17	15(68.2%)	4.00	0.45-35.5
		P for trend:	0.230			0.795			0.072	
*ALDH2* 487 *Glu/Lys*										
	N=44	N=26			N=10			N=16		
Low	19(43.2%)	9(34.6%)	1.00	Reference	5(50.0%)	1.00	Reference	8(50.0%)	1.00	Reference
Moderate	21(47.7%)	8(30.8%)	0.84	0.27-2.68	4(40.0%)	0.74	0.17-3.19	4(25.0%)	0.99	0.33-3.54
High	4(9.1%)	9(34.6%)	5.07	1.19-21.6	1(10.0%)	0.97	0.09-10.9	4(25.0%)	13.2	2.24-78.0
		P for trend:	0.057			0.817			0.004	
*ALDH2* 487 *Lys/Lys*										
	N=9	N=3			N=1			N=2		
Low	8(88.9%)	1(66.7%)	1.00	Reference	0(0.0%)	1.00	Reference	1(50.0%)	1.00	Reference
Moderate	1(11.1%)	2(33.3%)	16.0	0.65-390.9	1(100.0%)	NE*^5^		1(50.0%)	7.39	0.22-248.5
High	0(0.0%)	0(0.0%)	NE*^4^		0(0.0%)	NE*^5^		0(0.0%)	NE*^5^	
		P for trend:	0.090			NE*^5^			0.265	

Female	N=123*^2^	N=59			N=30			N=29		
*ALDH2* 487 *Glu/Glu*										
	N=61	N=28			N=17			N=11		
Low	46(75.4%)	20(71.4%)	1.00	Reference	13(76.5%)	1.00	Reference	7 (63.6%)	1.00	Reference
Moderate	12(19.7%)	6(21.4%)	1.32	0.42-4.15	3(17.7%)	0.97	0.22-4.34	3(27.3%)	1.69	0.37-7.75
High	3(4.9%)	2(7.1%)	2.75	0.37-20.8	1(5.9%)	2.30	0.18-29.1	1(9.1%)	2.44	0.18-32.8
		P for trend:	0.331			0.679			0.390	
*ALDH2* 487 *Glu/Lys*										
	N=51	N=28			N=12			N=16		
Low	45(88.2%)	26(92.9%)	1.00	Reference	12(100.0%)	1.00	Reference	14(87.5%)	1.00	Reference
Moderate	5(9.8%)	2(7.1%)	0.69	0.12-3.91	0(0.0%)	NE*^5^		2(12.5%)	1.26	0.20-7.58
High	1(2.0%)	0(0.0%)	NE*^5^		0(0.0%)	NE*^5^		0(0.0%)	NE*^5^	
		P for trend:	0.445			NE*^5^			0.891	
*ALDH2* 487 *Lys/Lys*										
	N=10	N=3			N=1			N=2		
Low	10(100.0%)	3(100.0%)	1.00	Reference	1(100.0%)	1.00	Reference	2(100.0%)	1.00	Reference
Moderate	0(0.0%)	0(33.3%)	NE*^5^		0(0.0%)	NE*^5^	0.58-569.7	0(0.0%)	NE*^5^	
High	0(0.0%)	0(0.0%)	NE*^5^		0(0.0%)	NE*^5^		0(0.0%)	NE*^5^	
		P for trend:	NE*^5^			NE*^5^			NE*^5^	

*^1^ For overall analyses, sex and age were adjusted. Age was adjusted in analyses for each sex.

*^2^ One controls was excluded from analysis because alcohol consumption data was not completed.

*^3^ One case was excluded from analysis because *ALDH2* genotype was not determined.

*^4^ Adjusted OR for only age because subjects’ sex for *Lys/Lys* genotype and moderate drinking was male.

*^5^ NE indicates not estimated because case or controls were absent in these categories.

To evaluate the prognostic effect of the *ALDH2* genotype among cases, we estimated the OR by the interval from diagnosis (incident group: ≤ 3 years; prevalent group; > 3 years). For the colorectal cancer, the ORs for having *487Lys* allele were 0.79 (0.49-1.27) and 0.93 (0.47-1.84) for the incident and prevalent groups, respectively. Similar analyses according to subsite also showed no difference between two groups.

## DISCUSSION

The present hospital based case-control study in Japan was conducted to evaluate the gene-environment interaction between alcohol and *ALDH2* polymorphism for the risk of colorectal cancer. We observed that cancer of the rectum was more influenced by alcohol consumption than colon cancer, with increased risk among individuals with a ‘weak’ *ALDH2* (*Glu/Lys*) genotype than those with the strongest (*Glu/Glu*) in terms of enzyme activity. This trend was obvious among men but not clear among women. The finding may be important with reference to differences in susceptibility to environment exposure among male individuals with different genetic traits.

Stronger harmful effects of alcohol drinking for *ALDH2 487Lss* allele carriers have been suggested for other cancers. Among Japanese alcoholics, the proportion of the *487Lys* allele carriers was reported to be higher for those with cancer than for those without cancer, especially for patients with oropharyngolaryngeal and esophageal cancers^16^^)^. We also found that a statistically significant interaction between heavy alcohol drinking and this *ALDH2* polymorphism^21^^)^. When the same amount of alcohol is drunk, the individuals with *Glu/Lys* genotype show a higher acetaldehyde concentration in serum and saliva than those with *Glu/Glu* genotype^23^^,^^24^^)^. Accordingly, it is very likely in biological sense that the harmful effect would be stronger in *487Lys* carriers drinking the same amount of alcohol.

To date, many epidemiological studies have evaluated the association between risk of colorectal cancers and alcohol consumption^2^^,^^3^^)^. Risk elevation being more consistently observed for rectum. However, for the Japanese, the difference in the OR of alcohol consumption between colon and rectal cancer has been controversial^25^^,^^26^^)^. Variation by subsite is supported by an animal experiment which demonstrated effects of alcohol only on rectum mucosa^8^^)^.

We observed an approximately 3 times higher OR for alcohol consumption among the male rectal cancer subjects with *Glu/Lys* compared to those with *Glu/Glu*. Although the estimated interaction (2.27) among male was not statistically significant, the positive value suggested that *Glu/Lys* genotype might enhance the effect of alcohol in high consumer males. To our knowledge, this is the first documentation of a possible existence of interaction and from the viewpoint of prevention, the results have important implication. The number of drinkers among females was very small in our subjects (Table 1, 3), the observed interaction between alcohol and *ALDH2* polymorphism may be interpretable only for male.

To date, two studies have concentrated attention on possible links between alcohol consumption and *ALDH2* polymorphism for the risk of colorectal cancer^16^^,^^17^^)^. Yokoyama et al evaluated the *ALDH2* among alcoholics (46 colon cancer patients and 487 non-cancer controls) and reported OR of 3.4 (1.5-7.5) for the heterozygotes (*Glu/Lys* compared with *Glu/Glu*)^16^^)^. Murata et al examined 265 colon cancer, 164 rectal cancer, and 121 non-cancer patients in the hospital-based prevalent case-control study. ORs of 2.13 (1.0-4.7) and 1.03 (0.5-2.2) were observed for *ALDH2 487Lys* allele carriers among colon and rectal cancers, respectively. Contrary to our result, the ORs for alcohol consuming were 3.1 (0.7-14.0) for colon cancer and 1.3 (0.2-7.0) for rectal cancer^17^^)^. The reason for the inconsistency is unclear, but random differences in alcohol consumption and *ALDH2* genotype distribution among controls might have affected the risk estimation.

We must say that there were several limitations to interpret this study results. Firstly, an attention needs to be paid to a prevalent case-control design. If the *ALDH2* genotype under study has a prognostic effect, the ORs derived from prevalent case-control design would be influenced^27^^)^. However, our result showed similar estimations for the *ALDH2* genotype, suggesting that the estimated ORs for *ALDH2* genotype and the interaction term were almost free from the prognostic effect. Secondly, the sample size for this study was not large enough to evaluate the interaction between alcohol consumption and *ALDH2* genotype, thus the results obtained from this study are not conclusive. Thirdly, in our analyses the dietary habits such as fiber or fat intake whose relations between colorectal cancer were suggested could not be analyzed. The interaction between these habits and alcohol consumption, it was not adjusted in this study, may affect the observed result.

In conclusion, the present study provided evidence for the possible existence of a gene-environment interaction between alcohol consumption and *ALDH2* genotype for the rectal cancer in Japanese. The prevention effect of alcohol reduction might be influenced by genotype of *ALDH2*. Although the interaction seems biologically plausible and analogous finding have been obtained for other sites of cancer, additional confirmatory studies are required with datasets for various ethnic groups.
